# On the lifespan of *Enchytraeus crypticus* - impact of iron (nanomaterial and salt) on aging

**DOI:** 10.18632/aging.206134

**Published:** 2024-10-24

**Authors:** Susana I.L. Gomes, Janeck J. Scott-Fordsmand, Mónica J.B. Amorim

**Affiliations:** 1 Department of Biology and CESAM, University of Aveiro, Aveiro 3810-193, Portugal; 2 Department of Ecoscience, Aarhus University, Aarhus DK-8000, Denmark

**Keywords:** long-term, aging, magnetite, nanobiomaterial, survival

## Abstract

Iron oxide nanomaterials (Fe_3_O_4_ NMs) have important biomedical and environmental applications, e.g. drug delivery, chemotherapy, magnetic resonance imaging contrast agents, etc. However, the environmental risks of such Fe_3_O_4_ are not fully assessed, particularly for soil living invertebrates, which are among the ones in the first line of exposure. Research has showed that longer-term exposure time is required to assess hazards of NMs, not predicted when based on shorter time and are therefore recommended. Thus, in the present study the effects of Fe_3_O_4_ NMs and FeCl_3_ were assessed in the terrestrial environment, using the soil model species *Enchytraeus crypticus* (Oligochaeta), throughout its entire lifespan (202 days). Two animals’ density were used: 1 (D1) and 40 (D40) animals per replicate, in LUFA 2.2 soil. The endpoints were survival and reproduction monitored over-time, up to 202 days. Results showed that density clearly affected the toxicity response, with higher toxicity and lower lifespan in D1 compared to D40. Overall, FeCl_3_ was more toxic than Fe_3_O_4_ NM in terms of reproduction, however, adult animals can be at higher (long-term) risk when exposed to Fe_3_O_4_ NM. Differences might be linked to slower Fe kinetics for the Fe_3_O_4_ NMs, i.e., slower Fe dissolution and release of ions.

## INTRODUCTION

The development of advanced materials like nanobiomaterials (NBMs) has attracted a lot of attention due to their potential to tackle some of the most demanding human-health related challenges such as cancer and new viruses. Among the potential NBMs, iron oxide nanomaterials/nanoparticles (NMs/NPs), particularly magnetite (Fe_3_O_4_), have been investigated due to their promising applications in the biomedical field and environmental remediation [1, 2]. In fact, formulations containing Fe_3_O_4_ NPs are already approved for use in magnetic resonance imaging contrast in Europe and United States of America [3]. Nevertheless, the potential risks of these materials are still under debate, particularly for environmental species, for which the studies are much scarcer [4].

The risk assessment and management of advanced materials is within the EU priorities and project calls, e.g. as within the EU H2020 BIORIMA project, where a framework for risk assessment and management of nano-biomaterials (NBMs) has been developed [5]. Within BIORIMA, one of the NBMs, the Fe_3_O_4_ NM coated with polyethyleneglycol and poly (lactic-co-glycolic acid) – PEG-PLGA has been thoroughly investigated. Low to no cytoxicity was reported to Fe_3_O_4_ NM-PEG-PLGA, for instance in the human colorectal carcinoma cell line HCT116 [6], in the human fibrosarcoma cell line (HT1080) and normal human fibroblast cells (BJ) [7], or to the fish RTgill-W1 cell line [8]. However, regarding ecotoxicity the few studies available employing Fe_3_O_4_ NMs (not the PEG-PLGA coated) indicated an overall low toxicity but changing toxicity. For example, in the water crustacean *Daphnia magna* the toxicity was low, but toxicity became higher with increasing organic matter content (e.g., 40% hatching reduction at 100 mg Fe_3_O_4_/L) [9]. A study with *Danio rerio* showed that Fe_3_O_4_ NPs accumulated in the fish, reaching a maximum concentration between 19 and 24 days, although being almost eliminated after 24 days in clear water [10]. There were no observed effects on fish’s survival at the tested concentrations: 4 and 10 mg Fe_3_O_4_ /L [10]. One study performed on the terrestrial snail *Cornu aspersum* and the fish *D. rerio* and *Carassius gibelio* showed that exposure to Fe_3_O_4_ NMs induced oxidative stress (increase in protein carbonylation) and damage (increase in lipid peroxidation), apoptosis (increase in Bax/Bcl-2 ratio, caspases levels and ubiquitin conjugates), and DNA damage to different tissues with the organisms [11]. Another study on a soil living invertebrate showed that the earthworm *Eisenia fetida* avoided the soil spiked with 1.5% Fe_3_O_4_ [12]. Further, Fe_3_O_4_ NMs caused oxidative stress (changes in catalase (CAT), peroxidase (POD) and superoxide dismutase (SOD) activities) and damage (increase in lipid peroxidation) to worms exposed via filter paper contact test up to 70 g Fe_3_O_4_/L [12]. Studies with several plant species, and soil microorganisms (mostly bacteria) revealed both positive and negative impacts of Fe_3_O_4_ NMs, as reviewed in [13], highlighting the uncertainty that remains regarding their environmental risks. Even though there is some information on the fate of Fe_3_O_4_ NMs over time, i.e., how it is affected by temperature, pH and organic matter [13], there is a clear lack of information on the long(er)-term toxic effects of those NMs in soils.

Enchytraeids are distributed in soils worldwide, contributing to improved soil structure and the degradation of organic matter [14]. They are also model species in soil ecotoxicology [15] and have been used in ecotoxicology laboratory tests for more than 50 years [14]. Recently a new test was developed covering the full lifespan of *Enchytraeus crypticus*, and while a much longer exposure is run it adds a new endpoint: longevity [16]. This test is particularly relevant to assessing the risks of NMs, for which effects have often been shown long-term and difficult to predict based on short-term tests [16–20]. Hence, in the present study we investigated the effects of a Fe_3_O_4_ NM, and compared it with FeCl_3_, throughout the lifespan of the soil invertebrate *E. crypticus* (Oligochaeta), ca. 202 days. The effects were assessed here in LUFA 2.2. soil, at two different animals’ density: 1 animal per replicate (D1) and 40 animals per replicate (D40). The endpoints were survival and reproduction over time.

## RESULTS

### Fe_3_O_4_ NM characterization

Fe_3_O_4_ NM revealed a strong agglomeration trend, despite the reported (by the producers) particle dimensions <200 nm (Table 1). Hydrodynamic diameter assessed by DLS showed large standard deviations of size and large PDI. The negative Z-potential values observed were lower (more negative) for the lower concentration, suggesting higher stability in water.

**Table 1:** Summary of characterization results from the Dynamic Light Scattering (DLS) on hydrodynamic diameter (Zeta average) and surface charge (Zeta potential) for Fe_3_O_4_ NM aqueous suspensions.

**Fe_3_O_4_ NM (mg/L)**	**hydrodynamic diameter Zeta-average (nm)**	**PDI**	**surface charge Zeta-potential (mV)**
400	4883 ± 750.2	1	-12.8 ± 0.44
200	5582 ± 1637	0.7	-22.1 ± 0.45

PDI, polydispersity index; AV, average; SD, standard deviation.

### Toxicity tests

For both test density, D1 (Density 1) and D40 (Density 40), the mortality in controls was less than 20%, within the validity criteria for the OECD standard enchytraeid reproduction test [15]. Soil pH varied between 4 (minimum) and 6 (maximum) for FeCl_3_ tests, and between 5 and 6 for Fe_3_O_4_ NM tests (for full details see Supplementary Table 1 and Supplementary Information).

**Figure 1:**
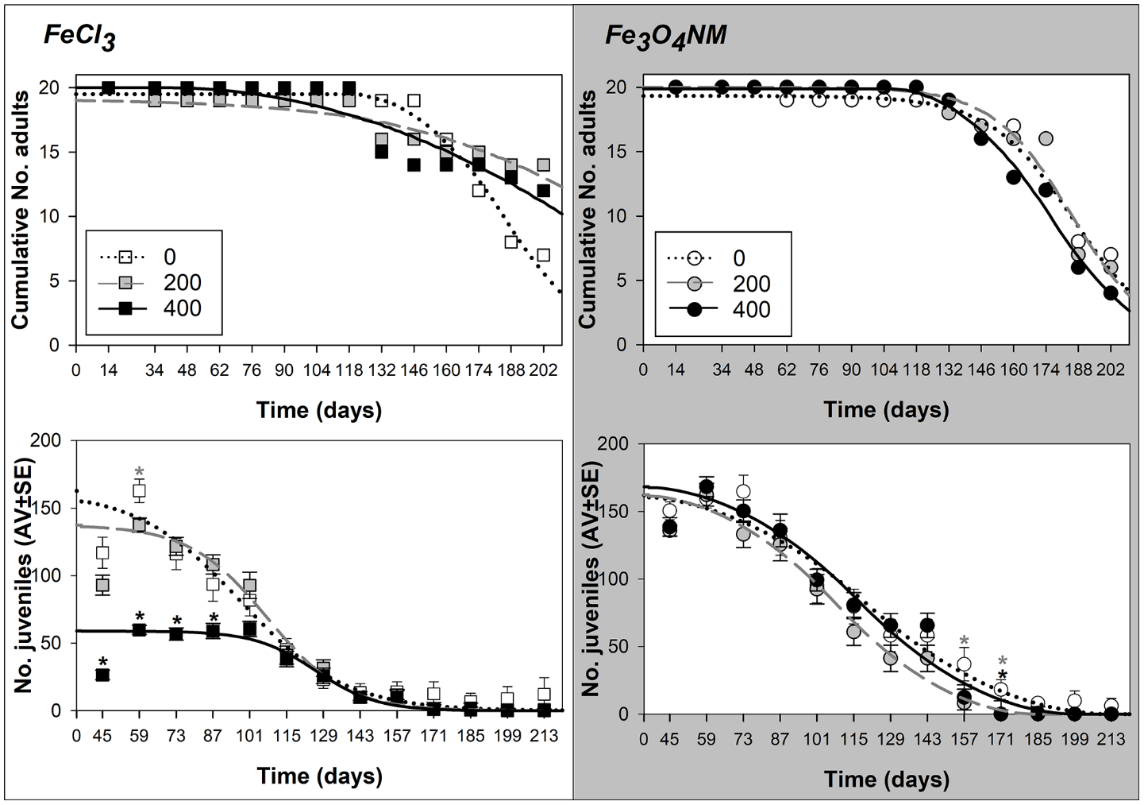
**Results from lifespan test with *Enchytraeus crypticus* when exposed to FeCl_3_ and Fe_3_O_4_ NM, in LUFA 2.2 soil, at the density of 1 adult organism per replicate, in terms of survival (top row: values expressed as cumulative number) and reproductive output (down row: values are expressed as average ± standard error).** Lines represent the models fit to data. *: p<0.05 (Dunnett’s), grey asterisk: 200 mg Fe/kg soil, black asterisk: 400 mg Fe/kg soil.

At D1, exposure to 200 mg FeCl_3_ increased enchytraeids lifespan in comparison to control (LT50=186, 237, and 212 days for 0, 200, and 400 mg Fe/kg soil of FeCl_3_, respectively, Table 2 and Supplementary 2). On the other hand, for Fe_3_O_4_ NM at 400 mg Fe/kg soil there was a slight negative impact on animals’ longevity (LT50=186, 185, and 175 days for 0, 200, and 400 mg Fe/kg soil of Fe_3_O_4_ NM, respectively) (Figure 1).

**Table 2:** Summary of the Effect Time (ETx, in days, with 95% confidence intervals - CI) for survival and reproduction (as number of juveniles/adult) for *Enchytraeus crypticus*, exposed to 0, 200, and 400 mg Fe/kg soil of FeCl_3_ and Fe_3_O_4_ NM, in LUFA 2.2 soil at two different organisms’ densities (1 organism (D1) and 40 organisms (D40)).

**Test material**		**FeCl_3_**		**Fe_3_O_4_NM**	
**Endpoint**	**Conc. (mg Fe/kg)**	**ET10 (95% CI)**	**ET50 (95% CI)**	**ET10 (95% CI)**	**ET50 (95% CI)**
*Density 1 (D1)*					
Survival	0	148 (141-156)	186 (182-189)	146 (137-155)	186 (182-190)
	200	135 (115-154)	237 (210-265)	148 (136-159)	185 (180-191)
	400	116 (95-138)	212 (191-232)	137 (131-143)	176 (173-179)
Reproduction	0	54 (40-67)	97 (92-104)	69 (53-84)	120 (113-127)
	200	75 (68-82)	108 (104-111)	66 (57-75)	109 (104-113)
	400	101 (92-110)	126 (122-130)	70 (57-83)	117 (111-123)
*Density 40 (D40)*					
Survival	0	n.e.	n.e.	212 (167-257)	258 (86-431)
	200	n.e.	n.e.	198 (190-206)	222 (185-259)
	400	n.e.	n.e.	n.e.	n.e.
Reproduction	0	41 (-25-108)	165 (140-189)	94 (56-133)	182 (161-203)
	200	58 (34-81)	126 (117-136)	71 (16-126)	194 (162-226)
	400	64 (43-86)	119 (109-128)	81 (49-113)	169 (153-184)

n.e.: no effect. For details on the models used and additional ET20 and ET80 values see Supplementary Table 2.

In terms of reproductive output, up to 101 days exposure, the EC50 was approximately 400 mg Fe/kg soil of FeCl_3_. After 115 days there were no differences between the 2 concentrations and control. However, the ET50 occurred latest for 400 mg Fe/kg soil (ET50=97, 108, and 126 days for 0, 200, and 400 mg Fe/kg soil of FeCl_3_, respectively). This increase with concentrations was even stronger at the ET10 level, see Table 2. For Fe_3_O_4_ NM, the differences between test treatments were overall small, but observed from day 73, with an earlier ET50 for 200 mg Fe/kg soil (ET50=120, 109, and 117 days for 0, 200, and 400 mg Fe/kg soil of Fe_3_O_4_ NM, respectively).

**Figure 2:**
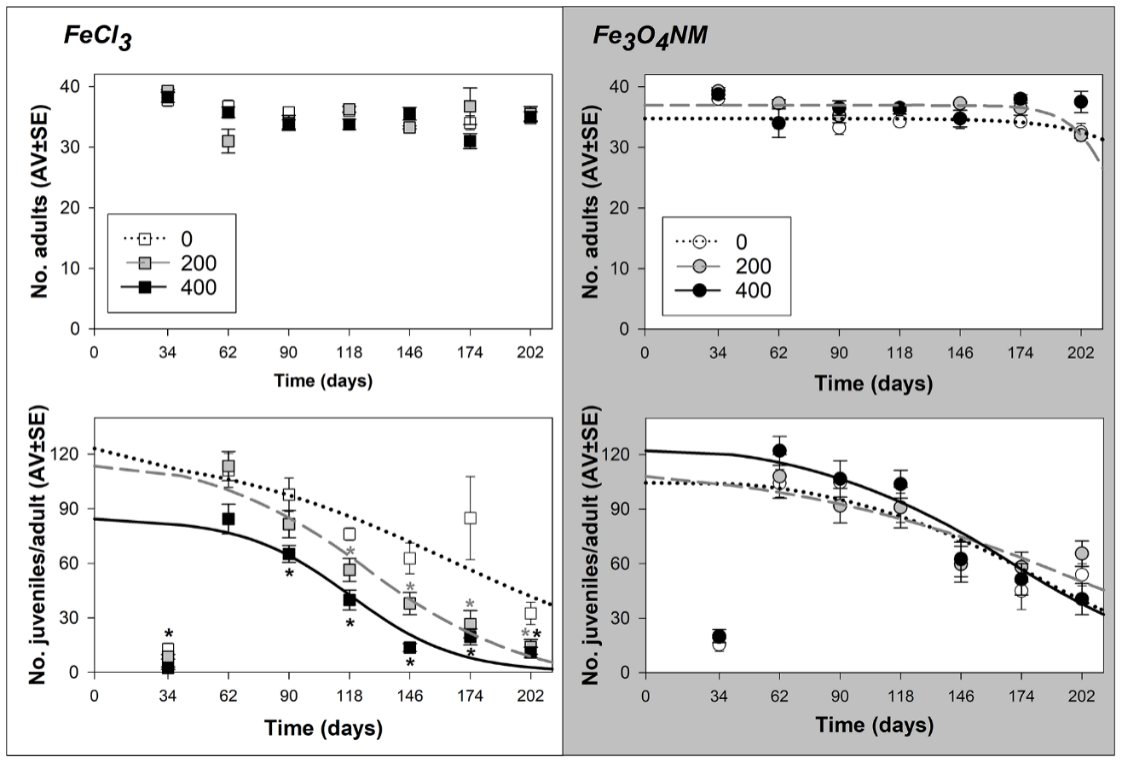
**Results from lifespan test with *Enchytraeus crypticus* when exposed to FeCl_3_ and Fe_3_O_4_ NM, in LUFA 2.2 soil, at the density of 40 adult organism per replicate, in terms of survival (top row) and reproductive output per surviving adult (down row).** All the values are expressed as average ± standard error. Lines represent the models fit to data. *: p<0.05 (Dunnett’s), grey asterisk: 200 mg Fe/kg soil, black asterisk: 400 mg Fe/kg soil.

At D40, adults survival was not affected (for the Fe_3_O_4_ NM test there is a tendency to decrease at 0 and 200 mg Fe/kg soil, at day 202) (Figure 2).

Reproductive effecst were determined based on the number of juvelines per surviving adult (Figure 2) for a direct comparison with D1 tests, but absolute number of juveniles are also reported in Supplementary Figure 1. In terms of reproductive output, there is a dose-dependent effect for FeCl_3_ (ET50=165, 126, and 119 days for 0, 200, and 400 mg Fe/kg soil of FeCl_3_, respectively). While for Fe_3_O_4_ NM there are no significant differences between test concentrations, the ET50 calculated (ET50=182, 194, and 169 days for 0, 200, and 400 mg Fe/kg soil of Fe_3_O_4_ NM, respectively) have overlapping confidence intervals (Table 2 and Supplementary Table 1).

## DISCUSSION

The survival of *E. crypticus* during its lifespan was comparable to previous [16]. That is in control conditions, at the density of 1 organism per replicate (D1), the control results (186 days) were similar to the previously reported [LT50=145 and 218 days from tests performed twice in [16]. As to reproduction, we previously observed a faster decline [ET50= 155 and 158 days [16], in comparison to 97 and 120 days in current results]. In Gonçalves et al. [16] two densities were also studied, D1 and D20, and the relative difference between the two densities in controls was similar, with shorter lifespan (lower ETx) for the lower density (D1) in comparison to higher density (D20) [16] or D40 (current results). The animals’ density clearly influenced the lifespan of *E. crypticus* and comparing the D20 [16] and D40 also indicated a further improvement in the longevity with increased density.

The interaction of animals’ density and toxicity was studied before in *E. crypticus* exposed to copper (Cu) [21, 22]. Their results showed that while density did not affect Cu toxicity in the one generation exposure [22], two-generation exposure revealed lower Cu toxicity for the higher density tested animals [21]. One of the proposed explanations is the higher likelihood of clustering behaviour of animals at higher densities, often observed when under stress, which indirectly lead to an exposure avoidance [21]. Although this will likely be a source of explanation in the toxicity, crowding must in general be beneficial for organisms as in control scenarios animals living alone had a shorter lifespan.

As to survival, for the D40 exposure none of the Fe forms caused effects within the 202 days, whereas at D1 a visible decline is observed, even if exposure to FeCl_3_ had a beneficial effect for elderly animals (lower mortality from 174 days old, compared to control). For Fe_3_O_4_ NM the opposite tendency was observed, as there was a negative effect at 400 mg Fe/kg soil (higher mortality after 146 days, compared to control). Based on the FeCl_3_ exposure results, we could hypothesize that increase in Fe availability would be beneficial for aged adults, e.g. because Fe is known to be a micronutrient, essential for many metabolic processes across living organisms (e.g., energy production, DNA repair and replication, regulation of gene expression, etc.). However, literature data suggests the opposite, i.e., Fe accumulation with age has been associated with many age-related diseases and lifespan shortening [23]. A shorter lifespan was in fact observed for Fe_3_O_4_ NM exposure (at 400 mg Fe/kg, D1). Different Fe kinetics from FeCl_3_ and Fe_3_O_4_ NM are expected in soils and must contribute to the differences [note that the soil was spiked at the beginning of the experiment and hence aged throughout the experiment duration]. Although to the best of our knowledge, the kinetics of FeCl_3_ or Fe_3_O_4_ NM have not been studied in soils, it has been shown that trivalent ions such as Fe^3+^ form strong complexes with humic substances (that comprise most of the dissolved natural organic matter in water bodies) [24]. In soils with FeCl_3_, the complexation of Fe^3+^ with the soils’ organic matter, would cause a decrease of Fe bioavailability over time, i.e. aging. In soils with Fe_3_O_4_ NM, Fe NMs probably provide a longer source of Fe^3+^ due to a slower/gradual NM dissolution and subsequent Fe^3+^ release. The slow release of Fe^3+^ seem to be supported by a concurrent experiment (unpublished project data) where not release was observed in 1h when measured in a BSA solution, i.e. Fe levels being below detection limit (ICP-OES - inductively coupled plasma optical emission spectrometer measurements). Obviously, the soil water conditions have an influence of the possible dissolution, for example it has been shown that media pH has a strong influence on Fe_3_O_4_ NM kinetics, with little to no dissolution reported in simulated body fluid with a pH of 7.4, and gradual dissolution in media with acidic pH [up to 60% dissolution in artificial endosomal fluid – pH 5.5, and 100% dissolution in artificial lysosomal fluid – pH 4.5] [25, 26]. Another study showed that synthesized Fe_3_O_4_ NM did not dissolve in distilled water, up to 3 weeks of storage, but dissolved in acidic media - citric and acetic acids [27]. The pH of the LUFA 2.2 soil spiked with Fe_3_O_4_ NM varied from 6.3 to 4.8 over time, thus the dissolution of Fe_3_O_4_ NM is likely to occur in time with Fe^3+^ release. This complex picture of bulk soil acidity is further mudded by the possible additional uptake of pristine Fe_3_O_4_ particles by the enchytraeids, with further intracellular dissolution (for instance in the gut and lysosomes) which contribute to toxicity.

In terms of reproduction, for D1 FeCl_3_ exposure, the age-related effects prevailed over the toxicity. Although at 400 mg Fe/kg soil, the number of juveniles is significantly lower than in control, the age-related decline occurs similarly from day 115 onwards to all exposures. This seems to indicate that FeCl_3_ exposure reduces the net reproduction of younger animals but does not induce a faster age-related decline (in comparison to control). For the D40 FeCl_3_ exposure, there was a dose-dependent age-related decline in reproduction (ET50: 0 > 200 > 400 mg Fe/kg soil), highlighting the importance of density on animals’ response to chemical exposure. Overall, the improved performance of enchytraeids at higher density (D40 compared to D1) allowed for a better discrimination of FeCl_3_ effects, i.e. dose-response.

For Fe_3_O_4_ NM exposure, the higher toxicity (lower reproduction ETx) at D1 in comparison to D40 is also observed. While at D40 there are no significant differences between test treatments (i.e., overlapping ETx), at D1, exposure to 200 mg Fe/kg soil resulted in a lower reproduction. Non-monotonic dose-responses have been reported before for NMs, in soil exposed animals, for instance to silver [28] and nickel [29]. Those findings were related to lower aggregation of NPs at lower concentrations, causing increased dissolution rates and/or higher number of single particles and hence higher toxicity. Several studies have shown that Fe_3_O_4_ NMs can undergo dissolution depending on pH (increased dissolution with decreasing pH) [25, 26]), or size (higher dissolution for larger particles [30]), and the processes are time dependent, reaching a plateau, in air atmosphere, at around 50 days [31]. However, none of those studies was performed in soil. The results here indicate that at 200 mg Fe/kg soil the Fe_3_O_4_ NM dissolved slightly more than at 400 mg Fe/kg soil, causing higher toxicity.

As mentioned, indications are that Fe kinetics play an important role in the differences observed between Fe_3_O_4_ NM and FeCl_3_. Moreover, it is likely that well-known aging related mechanisms, such as mTOR pathway [32], telomerase shortening [33], and/or insulin signaling [34] are differently affected by these Fe forms, hence, there is a toxicity due to release of Fe ion release. This should be further studied, e.g. via qPCR of target genes analysis, to capture regulations within aging mechanisms, as affected by exposure to Fe_3_O_4_ NM and FeCl_3_.

Overall, Fe_3_O_4_ NMs caused higher toxicity to enchytraeids throughout their full lifespan, than did FeCl_3_, for adult enchytraeids. Animals’ density clearly affected the responses, both regarding longevity and toxicity. Higher density (40 vs. 1 animal) was associated with longer lifespan and lower toxicity. In summary, the effects on aging as observed are due to the toxicity of Fe ion released and not on a perturbation of the aging mechanism alone.

## MATERIALS AND METHODS

### Test organism

The test species *Enchytraeus crypticus* Westheide and Graefe, 1992 was used. The cultures were kept in agar, consisting of Bacti-Agar medium (Oxoid, Agar No. 1) and a sterilized mixture of four different salt solutions at the final concentrations of 2 mM CaCl_2_·2H_2_O, 1 mM MgSO_4_, 0.08 mM KCl, and 0.75 mM NaHCO_3_, under controlled conditions of temperature (19 ± 1° C) and photoperiod (16:8 h light:dark). The cultures were fed with ground autoclaved oats twice per week. Juveniles of synchronized age (14-16 days after cocoon laying) were used for tests. For culture synchronization details see Bicho et al. [35].

### Test soil

The standard LUFA 2.2 natural soil (Speyer, Germany) was used. The soil main characteristics are: pH (0.01 M CaCl_2_) = 5.5, organic matter = 1.72%, CEC (cation exchange capacity) = 8.4 meq/100 g, WHC (water holding capacity) = 44.1%, grain size distribution of 10.7% clay (<0.002 mm), 15.7% silt (0.002–0.05 mm), and 73.6% sand (0.05–2.0 mm).

### Test materials and spiking

Iron (III) chloride hexahydrate (FeCl_3_.6H_2_O, 98-102%, puriss. p.a., ACS reagent, Sigma-Aldrich, USA) and Iron (II, III) oxide nanomaterial (Fe_3_O_4_, nanopowder, 50-100 nm particle size (SEM), 97% trace metals basis, Sigma-Aldrich) were used.

A similar concentration range was tested for both Fe materials: 0, 200, 400 mg Fe/kg soil dry weight (DW), which correspond to reproduction effect concentrations (EC)10 and EC20 for FeCl_3_ and no effect concentrations for Fe_3_O_4_ NM, based on previous study [36]. These are relevant as Fe is a common metallic element in natural soils and is also found in the form of magnetite (Fe_3_O_4_) [37, 38], including in the nanometer size range [37]. Predictions on the input of manufactured Fe_3_O_4_ NM are unknown but expected to increase due to their promising applications.

For FeCl_3_, an aqueous stock solution was prepared, serially diluted to the required concentrations, added to pre-moistened soil to reach 50% of the soil maximum WHC (maxWHC), and homogeneously mixed. The soil was spiked per batch, per concentration.

For Fe_3_O_4_ NM, the spiking followed the guidelines for solid/powder nanomaterials in soil [39]. In short, dry powders of the NM were mixed manually with dry soil to obtain the corresponding concentration range. After that, deionised water was added to reach 50% of the soil maxWHC, followed by thorough mixing. The soil was prepared per individual replicate for the density 40 (D40), and per batch per concentration-exposure period for the density 1 (D1). All the soil was spiked 7 days before test start, for both materials, being subject to aging over the test duration (up to 202 days).

### Fe_3_O_4_ NM characterization

Fe_3_O_4_ NM was characterized by Dynamic Light Scattering (DLS) and Zeta-Potential. The measurements were carried out with a Zeta-Sizer Malvern Instrument (Zetasizer Nano ZS, Malvern Ltd., UK) in backscattering mode to determine hydrodynamic size and charge (Zeta-potential). All measurements were performed in auto-mode at 25°C, with 3 consecutive measurements for each sample. The samples correspond to aqueous suspensions of Fe_3_O_4_ NM, prepared at the concentrations of 200 and 400 mg Fe/L.

### Test procedures

### Lifespan assays: survival and reproduction at density 1 (D1)

The lifespan assay at the density of 1 organism per replicate (D1) followed the procedures described by Gonçalves et al. [16]. One juvenile (14-16 days after cocoon laying) was placed in a petri dish (Ø 30mm) containing 5 g of moist soil (control or spiked). A total of 20 replicates per test condition was prepared (no sampling of replicates as in D40, as these continued exposure throughout the test). After 20 days (time for the organisms to grow and reach maturity), the adult organisms were transferred to new petri dishes of the same test condition. Every 14 days, the survival of the adults was recorded, and the surviving adults were transferred to new petri dishes. After each transfer, the previous test petri dishes were left for 11 more days to ensure that the cocoons laid had time to hatch; after that, the soil in the petri dishes was carefully transferred to glass vials, the juveniles were fixed with 96% ethanol and Bengal rose (1% in ethanol) and counted using a stereo microscope (Zeiss Stemi 2000-C). The test ran for a total of 202 days (plus 11 more days, again, to allow the cocoons from the last transfer to hatch), making up a total of 12 transfers (at days 34, 48, 62, 76, 90, 104, 118, 132, 146, 160, 174, and 188), at 20 ± 1° C and photoperiod of 16:8 h light:dark. Food (1 ± 0.2 mg grinded and autoclaved oats) was added at the test start and replenished weekly. Water was replenished weekly, based on weight loss.

### Lifespan assay: extra endpoints at density 40 (D40)

The performance of the lifespan assay at the density of 40 organisms per replicate (D40) was done as described above [16], with the following adaptations that include increased organisms’ density and sampling times. The density of 40 organisms per replicate was chosen to meet the necessary mass of organisms to be collected for further analysis. At test start, forty (40) juveniles of synchronized age (14-16 days after cocoon laying) were placed in a glass test vessel containing 40 g of soil (control or spiked). A total of 114 replicates per test condition were prepared (4 replicates for each sampling point). After 20 days, 4 replicates per test condition were sampled for future analysis. After collecting the organisms, the remaining adults and juveniles were counted, using a stereo microscope, to assess survival and reproduction, after fixation with ethanol and Bengal rose as described above. For the replicates that continue the exposure, the adult animals were transferred to new test vessels. Every 28 days, 4 replicates per test condition were sampled as described above. The test ran for a total of 202 days, making up a total of 6 transfers (at days 34, 62, 90, 118, 146, and 174), at 20 ± 1° C and photoperiod of 16:8 h light:dark. Food (33 ± 0.3 mg grinded and autoclaved oats) was added at the test start and weekly replenished. Water was replenished weekly, based on weight loss.

### Data analysis

One-way Analysis of Variance (ANOVA), followed by the post-hoc Dunnett’s method (for multiple comparisons), was used to assess the differences between test treatments and controls, at each sampling day, at a significance level of 0.05 (SigmaPlot 14.0) [survival at Density 1 not included because it represents cumulative numbers].

Effect Time (ETx) as time to reduce survival or reproduction in x%, were calculated modelling data to logistic or threshold sigmoid 2 parameters regression models, as reported in Supplementary Table 2, using the Toxicity Relationship Analysis Program software (TRAP v1.30a, USEPA). For reproduction data, the first time point (45 days for D1, and 34 days for D40) was not included for ETx calculations, because the time the animals had to reproduce was less than in the subsequent time points [the animals were not mature when the exposure started, but were mature for all the subsequent transfers], thus resulting in lower values and not a direct comparison.

## Supplementary Material

Supplementary Figure 1

Supplementary Tables
