# Supplementary material for: On the lifespan of Enchytraeus crypticus - impact of iron (nanomaterial and salt) on aging
Source: Aging (Albany NY). 2024 Oct 24;16(20):13012–24. doi: 10.18632/aging.206134 (PMC11552645; doi:10.18632/aging.206134)
Supplement: Supplementary Figure 1 [file aging-16-206134-s001.pdf]

SUPPLEMENTARY FIGURE

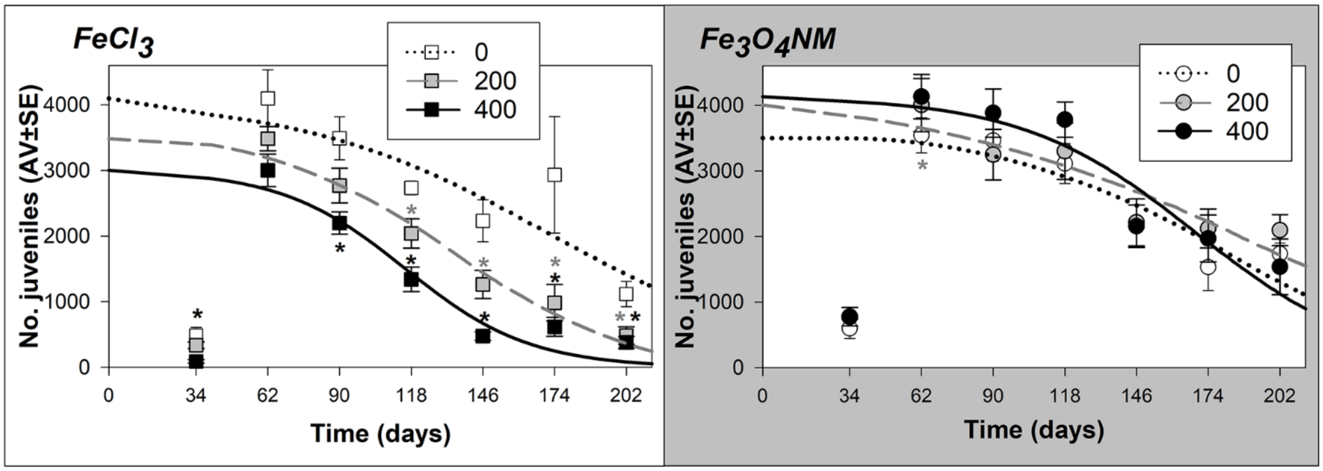

Supplementary Figure 1. Results from lifespan test with *Enchytraeus crypticus* when exposed to $FeCl_3$ and Fe_3O_4 NM, in LUFA 2.2 soil, at the density of 40 adult organism per replicate, in terms of reproductive output. The values are expressed as average ± standard error. Lines represent the models fit to data. *: $p<0.05$ (Dunnett´s), grey asterisk: 200 mg Fe/kg soil, black asterisk: 400 mg Fe/kg soil.
