# Supplementary material for: On the lifespan of Enchytraeus crypticus - impact of iron (nanomaterial and salt) on aging
Source: Aging (Albany NY). 2024 Oct 24;16(20):13012–24. doi: 10.18632/aging.206134 (PMC11552645; doi:10.18632/aging.206134)
Supplement: Supplementary Tables [file aging-16-206134-s002.pdf]

SUPPLEMENTARY TABLES

Supplementary Table 1. Results for pH measurements in soil spiked with $FeCl_3$ or Fe_3O_4 NM and aged over time (up to 202 days), for the test at density 1 (D1) and density 40 (D40).

	$FeCl_3$ (mg Fe/kg soil)			Fe_3O_4 NM (mg Fe/kg soil)		
**Time (days)**	**0**	**200**	**400**	**0**	**200**	**400**
Density 1 (D1)						
0	6.2	5.95	5.51	6.07	6.2	6.29
34	5.94	5.94	5.71	5.88	6.06	6.2
48	5.53	5.66	5.64	5.97	5.89	5.96
62	5.97	5.39	5.55	5.8	5.55	5.5
76	5.81	5.43	5.21	5.83	5.67	5.58
90	5.52	5.22	5.35	5.56	5.46	5.45
104	5.51	5.22	4.83	5.7	5.64	5.59
118	5.53	5.25	4.84	5.57	5.55	5.5
132	5.59	5.26	4.9	5.62	5.63	5.59
146	5.55	5.23	4.89	5.58	5.52	5.57
160	5.67	5.37	5	5.17	5.15	5.24
174	5.1	4.92	4.46	5.28	5.33	5.4
188	5.41	5.14	4.67	5.31	5.33	5.41
202	5.21	5.09	4.76	5.35	5.12	5.06
Density 40 (D40)						
0	6.01	5.47	5	6.16	5.98	5.9
34	5.96	5.47	5.08	6.17	5.89	5.82
62	4.92	5.11	4.96	5.19	5.55	5.29
90	4.78	4.51	4.93	5.56	5.09	4.86
118	5.08	4.59	4.77	5.5	4.86	4.74
146	4.94	4.61	4.27	5.6	5.31	5
174	4.26	4.41	4.1	5.31	4.96	4.84
202	5.37	5.07	4.85	5.35	5.03	4.88

Supplementary Table 2. Summary of the Effect Time (ETx, in days, with 95% confidence intervals - CI) for survival and reproduction (as number of juveniles/adult), for *Enchytraeus crypticus*, exposed to 0, 200, and 400 mg Fe/kg soil of $FeCl_3$ and Fe_3O_4 NM, in LUFA 2.2 soil at two different organisms' densities (1 organism (D1) and 40 organisms (D40)).

Material	Conc.	Endpoint	ET10 (95% CI)	ET20 (95% CI)	ET50 (95% CI)	ET80 (95% CI)	Model & parameters
Density 1 (D1)							
$FeCl_3$	0	Survival	148 (141-156)	161 (155-166)	186 (182-189)	203 (197-210)	Thres2P; S:1.48E-2, Y0:19.5, r2: 0.973
	200		135 (115-154)	173 (161-184)	237 (210-265)	302 (251-354)	Log2P; S:5.34E-3, Y0:19.125, r2:0.862
	400		116 (95-138)	148 (135-162)	212 (191-232)	257 (222-292)	Thres2P; S:5.79E-3, Y0:20, r2: 0.869
Fe_3O_4NM	0		146 (137-155)	161 (155-167)	186 (182-190)	212 (204-219)	Log2P; S:1.37E-2, Y0:19.333, r2:0.97
	200		148 (136-159)	162 (154-170)	185 (180-191)	209 (199-219)	Log2P; S:1.46E-2, Y0:20, r2:0.946
	400		137 (131-143)	150 (146-154)	176 (173-179)	195 (190-199)	Thres2P; S:1.42E-2, Y0:19.889, r2:0.988
$FeCl_3$	0	Reproduction	54 (40-67)	70 (60-80)	97 (92-104)	125 (116-134)	Log2P; S:1.26E-2, Y0:162.75, r2:0.966
	200		75 (68-82)	87 (82-92)	108 (104-111)	128 (123-133)	Log2P; S:1.69E-2, Y0: 137.42, r2:0.99
	400		101 (92-110)	110 (104-117)	126 (122-130)	142 (136-149)	Log2P; S:2.17E-2, Y0:59. r2:0.984
Fe_3O_4NM	0		69 (53-84)	86 (75-97)	120 (113-127)	145 (134-156)	Thres2P; S:1.07E-2, Y0:162.02, r2:0.965
	200		66 (57-75)	80 (74-87)	109 (104-113)	129 (123-135)	Thres2P; S:1.29E-2, Y0:162.35, r2:0.986
	400		70 (57-83)	86 (76-95)	117 (111-123)	139 (129-148)	Thres2P; S:1.19E-2, Y0:168.3, r2:0.972
Density 40 (D40)							
$FeCl_3$	0	Survival	n.e.	n.e.	n.e.	n.e.	
	200		n.e.	n.e.	n.e.	n.e.	
	400		n.e.	n.e.	n.e.	n.e.	
Fe_3O_4NM	0		212 (167-257)	229 (139-319)	258 (86-431)	288 (31-544)	Log2P; S:1.19E-2, Y0:34.75, r2:0.1
	200		198 (190-206)	207 (196-218)	222 (185-259)	237 (174-300)	Log2P; S:2.31E-2, Y0:36.958, r2:0.429
	400		n.e.	n.e.	n.e.	n.e.	
$FeCl_3$	0	Reproduction	41 (-25-108)	87 (44-129)	165 (140-189)	242 (184-301)	Log2P; S:4.46E-3, Y0:123, r2:0.516
	200		58 (34-81)	81 (64-97)	126 (117-136)	159 (144-174)	Thres2P; S:8.04E-3, Y0:113, r2:0.856
	400		64 (43-86)	84 (69-100)	119 (109-128)	153 (138-168)	Log2P; S: 1.02E-2, Y0: 84, r2:0.846
Fe_3O_4NM	0		94 (56-133)	124 (98-150)	182 (161-203)	224 (185-263)	Thres2P; S:6.32E-3, Y0: 105, r2:0.581
	200		71 (16-126)	112 (78-147)	194 (162-226)	252 (189-316)	Thres2P; S:4.52E-3, Y0: 108, r2:0.457
	400		81 (49-113)	110 (88-132)	169 (153-184)	210 (182-234)	Log2P; S:6.33E-3, Y0:122, r2:0.672

The models used are Logistic 2 parameters (Log2P) or Threshold sigmoid 2 parameters (Thres2P). S: slope; Y0: top point; n.e.: no effect.
